# Positive serum specific IgE has a short half-life in patients with penicillin allergy and reversal does not always indicate tolerance

**DOI:** 10.1186/2045-7022-4-34

**Published:** 2014-10-29

**Authors:** Janni Hjortlund, Charlotte Gotthard Mortz, Tore Bjerregaard Stage, Per Stahl Skov, Ronald Dahl, Carsten Bindslev-Jensen

**Affiliations:** Department of Dermatology and Allergy Centre, Odense University Hospital, Sdr. Boulevard 29, DK-5000 Odense C, Denmark; Clinical Pharmacology, Institute of University of Southern Denmark, Odense C, Denmark

**Keywords:** Penicillin allergy, 7-day oral challenge, Prolonged challenge, Specific IgE to penicillin, Half-life

## Abstract

**Background:**

The positive and negative predictive values of specific IgE to penicillins are not well established for penicillin hypersensitivity. One reason may be that serum IgE levels to penicillin diminish over time. The objective in this study was to investigate variations in serum half-life (T½) for specific IgE to penicillins (s-IgE) and to evaluate the outcome of penicillin challenges in patients with previous but not present specific IgE to penicillins.

**Methods:**

Two subgroups were investigated. All included patients had a history of penicillin allergy with reported symptoms such as urticaria/angioedema or unclassified cutaneous rash. T½ of specific IgE to penicillins was calculated based on sera from 29 patients with repeated measurements of s-IgE. Twenty-two patients with a previous positive s-IgE was followed and challenged with penicillin when IgE had become negative.

**Results:**

The T½ for s-IgE varied between the 26 patients with decreasing s-IgE from 1.6 months to 76.4 months and 52% had a T½ of less than a year. The three patients with stable and increasing IgE-values showed T½ approaching infinity A total of 29 challenges with β-lactams were performed. Four different patterns were seen when evaluating the clinical reaction to challenge (positive/negative) and post-challenge boost of s-IgE (yes/no). Eight (36.4%) had negative challenge and negative post-challenge s-IgE, eight (36.4%) negative challenge, but positive post-challenge s-IgE levels. 3 (13.6%) had positive challenge and positive post-challenge s-IgE whereas 3 (13.6%) were challenge positive, but had negative post-challenge s-IgE.

**Conclusion:**

Specific IgE to penicillins declines over time stressing the importance of a close time relation between diagnostic work-up and clinical reaction. Reversal of previously positive s-IgE may still be associated with positive penicillin challenges and/or re-boostering of s-IgE to positivity.

## Background

Correct diagnosis of allergy to penicillin is of great importance, but in contrast to what is found in a patient with a history of grass pollen allergy, discrepancy between skin test, measurement of specific IgE and outcome of challenge is often seen in patients with suspected penicillin allergy. Recommendations to patients based on false-negative results can lead to severe adverse reactions whereas false-positive results can lead to administration of less effective and more expensive drugs. We have previously demonstrated [1, 2] that challenge tests with penicillin play an important role in the diagnosis of hypersensitivity reactions to penicillin. Including a 7-day treatment with a therapeutic dose of penicillin is of special importance because patients with negative skin test and negative specific IgE to penicillins, but with a convincing history of penicillin allergy, may be positive in such a prolonged challenge test with therapeutic dosing for 7 days. International guidelines (ENDA and ICON) [3–6] only propose a single dose challenge. The positive and negative predictive values of serum specific IgE to penicillins (s-IgE) are not well established [7–9]. One reason may be that s-IgE levels to penicillin diminish over time [9–12]. One of the aims of this study was to determine the serum half-life (T½) of s-IgE. The second aim was to assess the clinical importance of previous IgE sensitization in patients, where s-IgE (penicillin V and G, ampicillin and amoxicillin) had become negative. To our knowledge a study on penicillin challenges has never been performed in patients with previous, but not present measurable values of specific IgE to penicillins.

## Methods

### Patients

In the period September 2003 to October 2013, our serum bank contains samples from 2791 adult patients with suspected penicillin allergy, who had performed measurements of s-IgE to penicillins. Hereof a total of 287 adult patients had a positive s-IgE to one or more penicillins (benzylpenicillin (pen G), phenoxymethylpenicillin (pen V), ampicillin (AMP), amoxicillin (AX)).

#### Serum elimination half-life (T½) of s-IgE (pen G, V, AMP, AX)

**Figure 1:**
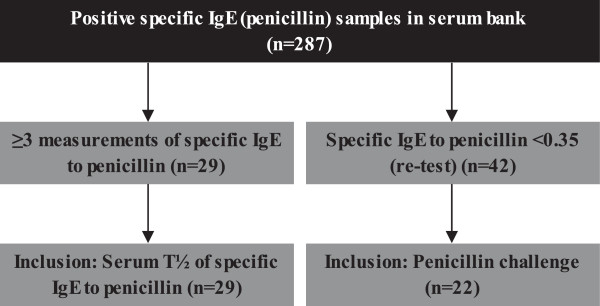
**Algorithm for inclusion criteria.** 29 patients included for the estimation of T½ of specific IgE to penicillins. 22 patients included in penicillin challenge in patients with previous IgE sensitization to penicillins. A total of 50 individuals were included, since 3 subjects were included in both the penicillin challenge in patients with previous IgE sensitization to penicillin and in the estimation of T½ of specific IgE to penicillins. IgE: Immunoglobulin E, T1/2: half-life for specific IgE to penicillins.

A total of 29 patients (10 men and 19 women) had 3 or more measurements of s-IgE over a period of up to 59 months. These were included for determination of T½ of s-IgE (pen G, V, AMP, AX), Figure 1. The median level of s-IgE measurements was 3 measurements (range 3 – 15 measurements). There were no demographic differences between the patients included and the patients with less than three IgE-measurements not included. Further no demographic differences were found between the patients with positive s-IgE and the patients with negative s-IgE (p > 0.05). The most frequently reported symptoms were urticaria and/or angioedema (17 cases, 58.6%), unclassified cutaneous rash (11 cases, 37.9%) and anaphylaxis (1 case, 3.5%). Pen V was the culprit drug in most cases, 21 (72.4%), the remaining reacting to dicloxacillin (DX) (4 cases, 13.8%), AX (3 cases, 10.3%) and unknown in one case (3.5%).

#### Penicillin challenge in patients with previous positive specific IgE to penicillins

The serum bank contained sera from forty-two patients followed prospectively with repeated measurements of s-IgE that had reverted to negative test results. Of these twenty-two (12 men and 10 women) agreed to participate in the present study. All patients had negative skin prick test (SPT) and intracutaneous test (ICT) to penicillins prior to penicillin challenge, Figure 1.

The β-lactam eliciting the initial reaction (culprit drug) was pen V in 15 cases (62.5%), AX in 5 (20.8%) and DX in 2 (8.3%).

According to the history reactions commencing within 1 h after drug intake were classified as immediate reactions (IR) and occurred in 7 patients. Reactions commencing 1 h or longer after drug intake were classified as nonimmediate reactions (NIR) and seen in 15 patients. Twelve of the 22 patients reported urticaria and/or angioedema (6 IR, 6 NIR) and 10 patients with unclassified cutaneous rash (1 IR, 9 NIR). Sixteen of the 22 patients had experienced their initial clinical reaction within one year prior to measurement of the positive s-IgE (thirteen even within 6 months), two within 1–5 years and 4 more than five years prior to blood sampling.

**Figure 2:**
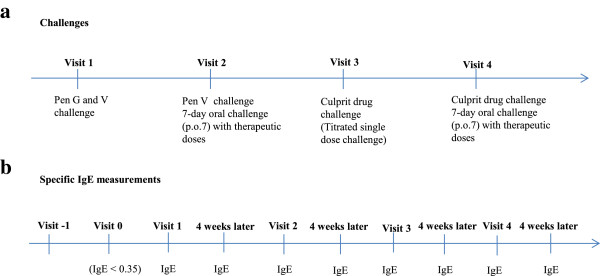
**Challenge program in the patients with previous positive specific IgE to penicillins. (A)** Challenge program within each visit. **(B)** Program for measurements of specific IgE to penicillins. In continuation of each challenge, specific IgE measurements were performed and repeated 4 weeks later. In case of any positive clinical reaction or renewed positive specific IgE, further challenges were terminated. Visit -1: The visit of the first positive measurement of specific IgE to penicillin. Visit 0: The visit of the first negative measurement of specific IgE to penicillin. Pen G: Benzylpenicillin, Pen V: Phenoxymethylpenicillin, p.o.7: prolonged oral challenge with penicillin for 7 days, IgE: specific immunoglobulin E to penicillins (Pen G, Pen V, ampicillin, amoxicillin). All patients had negative specific IgE to penicillin at the time of penicillin challenge (visit 0 and 1).

All patients showed undetectable levels of s-IgE at the time of penicillin challenge. The challenge tests were performed according to procedures in our two previous studies [1, 2], with the modifications described in Figure 2a + 2b. At visit 1, a titrated challenge with penicillin was performed by intravenous injection of pen G from 100 IE to 1.000.000 IE in 10-fold increments and 20 min interval, followed by administration of a single oral dose of 400 mg pen V 20 min after the last i.v. dose. If negative, the patient was at visit 2 given the first two doses (80 mg followed by 800 mg 30 min later) of a 7-day course of pen V in the clinic. If this challenge was negative, the patient continued with 800 mg t.i.d. at home. If the above challenges with pen G and V proved negative and if the case history or previous positive specific IgE pointed to a reaction to a different β-lactam antibiotic, the patient was subsequently challenged with the culprit drug (AX, AMP, DX) in a titrated oral protocol (visit 3). Three doses were administered with 30-min. interval (5, 50 and 500 mg). If negative, the patient was at visit 4 given the first dose of a 7-day course of culprit drug (500 mg) followed by dosing (500 mg b.i.d. (AMP, AX)), and (500 mg t.i.d. (DX)) at home. In case of a positive reaction (positive challenge or positive s-IgE) further challenges were terminated. Blood samples for determination of s-IgE were drawn at visit −1, 0, 1, 2, 3, 4 and four weeks after each visit. In case of renewed positive s-IgE after any of the visits, the investigation was stopped and the patient was followed with measurement of s-IgE with 3–6 months interval.

The Regional Scientific Ethical Committees for Southern Denmark has approved the study (approval number S-20100091). All patients gave written informed consent for the study as well as consenting to storage of their blood in the serum bank.

### Measurement of specific IgE to penicillins (s-IgE)

S-IgE levels to pen G, pen V, AMP, AX and MDM (minor determinant mix) were measured using ImmunoCAP (ThermoFisher Scientific, Uppsala, Sweden). A positive result was defined as a value ≥ 0.35 kU/L. Total IgE levels were measured in 20 of the 22 patients included in the penicillin challenge in patients with previous IgE sensitization to penicillins (ThermoFischer Scientific, Uppsala, Sweden).

#### Serum elimination half-life (T½) of s-IgE (pen G, V, AMP, AX)

Determination of the elimination half-life (T½) of s-IgE was calculated using WinNonlin 6.3 (Pharsight, Mountain View, CA, USA). T½ was calculated by the following equation: T½ = ln [2]/*k*, where *k* is the positive value of the terminal slope of the IgE concentration-time curve. The majority of the patients (n = 20) had experienced their initial clinical reaction within one year prior to measurement of the positive s-IgE, three within 5 years and 6 more than five years prior to blood sampling.

### Statistics

Data were analyzed for significance using the Fischer’s exact test. Probability values of < 0.05 were considered statistically significant. The statistical analyses were performed with STATA 13, StataCorp, College Station, Texas, USA.

## Results

The study included 51 cases (48 individual patients, where 3 subjects (all men) were included in both the penicillin challenge in patients with previous IgE sensitization to penicillin and in the estimation of T½ of s-IgE.

### Serum elimination half-life (T½) of s-IgE (pen G, V, AMP, AX)

**Figure 3:**
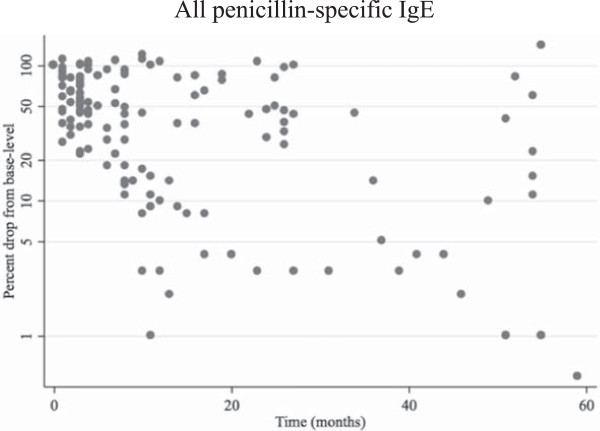
**Relative changes in serum concentrations of specific IgE to penicillin presented as the change in amount of specific IgE to penicillin from base line.** The level of the patients’ first positive s-IgE measurement is set to 100 % on a log-scale. Time of sampling is presented in months from the patients’ first positive s-IgE value. All penicillin – specific IgE includes benzylpenicillin, phenoxymethyl penicillin, ampicillin and amoxicillin.

**Figure 4:**
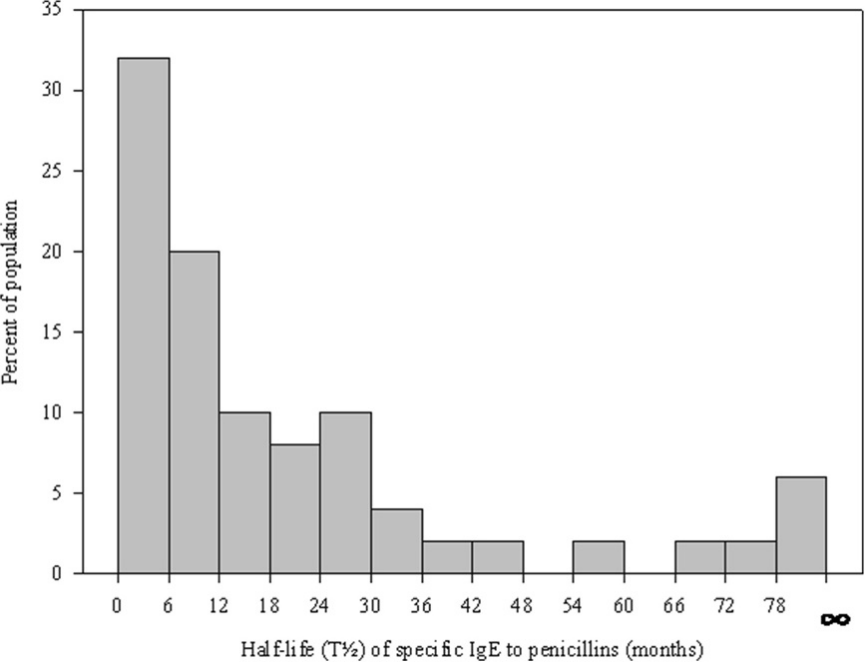
**Distribution of the estimated serum half-life (T½) of specific IgE to penicillins.** No differences were seen between the four penicillins. ∞: Symbol of infinity, including the 3 cases with stable and increasing IgE-values over time. Specific IgE to penicillins includes benzylpenicillin, phenoxymethyl penicillin, ampicillin and amoxicillin.

For estimation of T½ of s-IgE, 29 (19 women + 10 men) patients with three or more consecutively serum measurements of specific IgE to one or more penicillins were included. Median level (25–75 percentiles) for s-IgE at inclusion was 4.3 kU/l (1.4-18.5). Figure 3 shows a scatter-plot of the sequential positive IgE values for all penicillins, where the values are given as percentage of the initial value. A total of 189 measurements of s-IgE were evaluated; 27 initial values of positive specific IgE to pen V (17 with mono-sensitization), 9 to initial pen G, 10 to initial AMP (2 with mono-sensitization) and 4 to initial AX. In three cases initial specific IgE to all four penicillins were positive. In 8 cases, the specific IgE level was stable over time (up to 55 months). The T½ for s-IgE between the 26 patients with decreasing s-IgE varied from 1.6 months to 76.4 months. The three patients with stable and increasing IgE-values showed T½ approaching infinity, Figure 4. T½ was less than 6 months in 32% of the patients, 52% was less than a year and 84% had a T½ of less than 3 years.

### Penicillin challenge in patients with previous positive specific IgE to penicillins

Twenty-two patients had a previous positive s-IgE; all were skin test negative to the four penicillins (data not shown). The demographic data, results of specific IgE determination and outcome of their challenge(s) are presented in Table 1. Six of the 22 patients (no. 1–6) had a positive reaction during one of the challenges, three after a single dose (no. 1, 2 and 5), the remaining reacted during (no. 4 and 6) or after (no. 3) the 7-day oral challenge. In 3 of the 6 patients an elevated post-challenge s-IgE was detected. Sixteen patients (no. 7–22) were negative in challenges; in eight of these, (no. 7–14), positive post-challenge s-IgE was observed either after single dose (n = 3) or 7-day oral (n = 5) challenge. Post-challenge s-IgE levels showed large individual variation, neither related to initial s-IgE level nor to positive or negative outcome of the challenge, Table 1. Eleven patients had measurable post-challenge s-IgE, (no. 1, 3, 5, 7–9, 10–14); 3 with positive clinical reaction in challenge (2 IR, 1 NIR) and 8 negative (5 IR, 3 NIR); they were subsequently followed with repeated blood samples as described in Materials and Methods. The post-challenge s-IgE levels declined over time with varying rates, (data not shown). Five patients are currently IgE negative (no. 1, 3, 5, 9, 11), six are still positive (no. 7, 8, 10, 12–14), but specific IgE is declining. The relation between symptoms and signs in case history and during challenge together with post-challenge s-IgE is presented in Table 2. Three patients (no. 7–9) had negative i.v/single dose challenge with penicillin (G and V) but had positive post-challenge s-IgE to pen V four weeks later and therefore further challenge was cancelled. In all three subjects pen V was the culprit drug. Five patients (no. 10–14) showed positive post-challenge s-IgE four weeks after a negative outcome of 7-day oral challenge with pen V. In all 5 cases pen V was the culprit drug and all had initial positive levels of specific IgE to pen V. In three of the patients (no. 11, 13 and 14), post-challenge s-IgE to other penicillins developed. Eleven patients with either another culprit drug than pen G or V (no. 5, 6 (DX), 16–18, 20, 21 (AX)) or positive specific IgE to AMP (no. 5, 6, 9, 14, 15, 19) were also challenged with the culprit drug in case of negative penicillin challenge and negative post-challenge s-IgE (n = 10, no. 9, 14 not challenged, Table 1). Of these, only the two patients with DX as culprit drug (no. 5, 6) were positive in challenge. Post-challenge level of s-IgE was neither associated with single dose nor 7-day oral challenge outcome, Table 3.

**Table 1:** **Demographic data and clinical results of the 22 patients with a history of penicillin allergy and with initial positive serum specific IgE to penicillin**

Patient data*				Primary reaction		Initial IgE						Iv/single dose challenge					Post IgE (4 w after challenge)				Challenge p.o.7				Post IgE (4 w after challenge)			
		Age (years)																										
No	Sex	IgE pos	IgE neg	Culprit drug	Type of reaction	Total	Pen V	Pen G	AMP	AX	MDM	Pen G	Pen V	AMP	AX	DX	Pen V	Pen G	AMP	AX	Pen V	AMP	AX	DX	Pen V	Pen G	AMP	AX
1	F	34	35	Penicillin V	urt (IR)	53.1	**0.9**	0	0	0	0	Neg	**Pos**	NT	NT	NT	**2.8**	0	0	0	\	NT	NT	NT	NT	NT	NT	NT
2	M	65	69	Penicillin V	urt (NIR)	248.6	**0.4**	0	0	0	0	Neg	**Pos**	NT	NT	NT	0	0	0	0	\	NT	NT	NT	NT	NT	NT	NT
3	M	38	38	Penicillin V	urt+angio (NIR)	101.5	**0.5**	0	0	0	0	Neg	Neg	NT	NT	NT	0	0	0	0	**Pos**	NT	NT	NT	**0.4**	0	0	0
4	M	18	19	Penicillin V	urt (NIR)	54.1	**0.4**	0	0	0	0	Neg	Neg	NT	NT	NT	0	0	0	0	**Pos**	NT	NT	NT	0	0	0	0
5	M	56	57	Dicloxacillin	urt+angio (IR)	271.7	**0.5**	**0.4**	**0.8**	0	0	NT	NT	NT	NT	**Pos**	**0.4**	0	**0.4**	0	\	NT	NT	NT	NT	NT	NT	NT
6	M	77	81	Dicloxacillin	rash (NIR)	1773.0	0	0	**0.7**	0	0	Neg	Neg	NT	NT	Neg	0	0	0	0	Neg	NT	NT	**Pos**	0	0	0	0
7	F	30	34	Penicillin V	urt (IR)	4.2	**0.9**	**0.8**	0	0	0	Neg	Neg	NT	NT	NT	**1.2**	**0.5**	0	0	\	NT	NT	NT	NT	NT	NT	NT
8	M	30	33	Penicillin V	urt (IR)	26.8	**0.4**	0	0	0	0	Neg	Neg	NT	NT	NT	**0.8**	0	0	0	\	NT	NT	NT	NT	NT	NT	NT
9	M	51	56	Penicillin V	rash (NIR)	580.7	0	0	**0.4**	0	0	Neg	Neg	NT	NT	NT	**0.5**	**0.7**	0	0	\	NT	NT	NT	NT	NT	NT	NT
10	M	43	43	Penicillin V	rash (IR)	10.2	**0.4**	0	0	0	0	Neg	Neg	NT	NT	NT	0	0	0	0	Neg	NT	NT	NT	**>100**	0	0	0
11	M	58	58	Penicillin V	urt (IR)	142.7	**0.4**	0	0	0	0	Neg	Neg	NT	NT	NT	0	0	0	0	Neg	NT	NT	NT	**1.5**	**0.4**	0	0
12	F	48	49	Penicillin V	urt+angio (IR)	38.3	**0.8**	0	0	0	**2.3**	Neg	Neg	NT	NT	NT	0	0	0	0	Neg	NT	NT	NT	**3.9**	0	0	0
13	M	51	55	Penicillin V	rash (NIR)	363.7	**0.4**	0	0	0	0	Neg	Neg	NT	NT	NT	NT	NT	NT	NT	Neg	NT	NT	NT	**4.3**	**0.4**	**0.4**	0
14	F	46	49	Penicillin V	urt+angio (NIR)	4.3	**3.4**	**1.4**	**0.5**	**0**	0	Neg	Neg	NT	NT	NT	0	0	0	0	Neg	NT	NT	NT	**9.8**	**2.8**	**2.9**	**1.3**
15	F	34	38	Penicillin V	rash (NIR)	491.2	0	0	**0.9**	0	0	Neg	Neg	Neg	NT	NT	0	0	0	0	Neg	Neg	NT	NT	0	0	0	0
16	F	63	67	Amoxicillin	rash (NIR)	107.3	**0.4**	0	0	0	0	Neg	Neg	NT	Neg	NT	0	0	0	0	Neg	NT	Neg	NT	0	0	0	0
17	M	60	61	Amoxicillin	rash (NIR)	698.1	**3.2**	0	0	0	0	Neg	Neg	NT	NT	NT	0	0	0	0	Neg	NT	Neg	NT	0	0	0	0
18	F	60	67	Amoxicillin	urt (NIR)	NT	**0.5**	0	0	0	0	Neg	Neg	NT	Neg	NT	0	0	0	0	NT	NT	Neg	NT	0	0	0	0
19	M	52	52	Penicillin V	rash (NIR)	NT	0	0	**0.7**	0	0	Neg	Neg	Neg	NT	NT	NT	NT	NT	NT	Neg	Neg	NT	NT	NT	NT	NT	NT
20	F	56	61	Amoxicillin	rash (NIR)	NT	**1.1**	**1.0**	0	0	0	Neg	Neg	NT	Neg	NT	NT	NT	NT	NT	Neg	NT	Neg	NT	NT	NT	NT	NT
21	F	58	59	Amoxicillin	rash (NIR)	NT	**0.4**	0	0	0	0	Neg	Neg	NT	Neg	NT	NT	NT	NT	NT	Neg	NT	Neg	NT	NT	NT	NT	NT
22	F	35	43	Penicillin V	urt (NIR)	355.8	**0.4**	0	0	0	0	Neg	Neg	NT	NT	NT	NT	NT	NT	NT	Neg	NT	NT	NT	NT	NT	NT	NT

*Patient data are illustrating the patients’ age at the time of respective IgE positive and IgE negative results.

F: female, M: male, urt: urticarial, angio: angioedema, p.o.7: 7-day oral challenge, pos: positive challenge, neg: negative challenge, NT: not tested, MDM: Minor determinant mix for penicillin, \: not tested due to either positive i.v./single dose challenge or to positive post-challenge specific IgE to penicillin, bold data: positive result, 0: negative result.

**Table 2:** **Relation between clinical reaction (case history) and reaction at penicillin challenge, eliciting penicillin, challenge type and post-challenge levels of s-IgE**

	Type of reaction		Type of challenge	Post-challenge s-IgE
Patient no.	Case history	Challenge	Positive	4 weeks later
1	**Urticaria (IR)**	**Urticaria (IR)**	Single p.o Pen V	Positive
2	Urticaria (NIR)	Maculopapular rash (NIR)	Single p.o Pen V	Negative
3	**Urticaria/angioedema (NIR)**	**Urticaria (NIR)**	P.o.7 Pen V	Positive
4	**Urticaria (NIR)**	**Urticaria (NIR)**	P.o.7 Pen V	Negative
5	**Urticaria/angioedema (IR)**	**Urticaria (IR)**	P.o.7 DX	Positive
6	Urticaria (NIR)	Maculopapular rash (NIR)	P.o.7 DX	Negative

Bold text indicate concordance between type of reactions eliciting primary reaction and challenge. In the three cases of positive post-challenge s-IgE (no. 1, 3, 5) concordance was found.

In all cases, the culprit drug and the drug eliciting the clinical reaction during challenge were identical (see Table 1).

IR: immediate reaction, NIR: nonimmediate reaction, s-IgE: serum specific IgE to penicillin, p.o.7: 7-day oral challenge, p.o: per oral, Pen V: phenoxymethylpenicillin, DX: dicloxacillin.

**Table 3:** **Specific IgE level (positive/negative) compared to outcome of penicillin challenge (positive/negative)**

		I.v/single p.o (pen V,G) (n = 21)**		P.o.7 (pen V) (n = 15)*		Single p.o (other) (n = 8)*		P.o.7 (other) (n = 8)*	
		Pos (n = 2)	Neg (n = 19)	Pos (n = 2)	Neg (n = 13)	Pos (n = 1)	Neg (n = 7)	Pos (n = 1)	Neg (n = 7)
**Post-challenge s-IgE**	**Pos (n = 11)**	1	3	1	5	1**	0	0	0
	**Neg (n = 25)**	1	11*	1	4	0	4	1	4*
	**NT (n = 12)**	0	5	0	4	0	3	0	3

A total of 21 patients were challenged with i.v/single p.o pen V, followed by p.o.7 pen V in 15 patients. **Patient no. 5 (Table 1) was only challenged with culprit drug (DX).

Nine patients also challenged with other drug (either their culprit drug or because of previous positive specific IgE to AMP or AX). In 8 cases initiated by single p.o, and in 1 case* (no. 17, Table 1) initiated by challenge with pen G and V (i.v/single p.o), followed by challenge with AX (culprit drug) in p.o.7 (not preceded by single p.o AX). No. 17, Table 1 had not performed p.o.7 with pen V.

s-IgE: serum specific IgE to penicillin, I.v: intravenous, Single p.o: single dose oral challenge, P.o.7: 7-day oral challenge, Pen V: phenoxymethylpenicillin, Pen G: benzylpenicillin, DX: dicloxacillin, AX: amocxicillin, Pos: positive, Neg: negative, NT: not tested.

## Discussion

We describe for the first time the outcome of penicillin challenge in patients with a history of penicillin allergy and with previous but not present measurable levels of specific IgE to penicillins. Further, we describe for the first time variations in serum half-life of specific IgE in patients, sensitized to a penicillin.

### Serum elimination half-life (T½) of s-IgE (pen G, V, AMP, AX)

The present study confirms that levels of s-IgE decline over time with different rates in the single patient, neither related to initial level nor to positivity of reaction. Possible underlying mechanisms behind this variable decay is unknown, but in concordance with previous studies having shown that specific IgE antibody levels tend to decrease over time in the absence of new contact with the hapten [9–12]. A decline in IgE antibody responses has previously been described for other allergens such as chlorhexidine [13] and insect venom [14]. The patients in our study had no known exposure to a penicillin after the initial reaction.

T½ for s-IgE varied from 1.6 to 76.4 months. In approximately half of the patients T½ was less than a year. Our results thus imply, that a considerable number of penicillin allergic patients will present with a negative s-IgE depending on the time elapsed since their clinical reaction had taken place.

#### Penicillin challenge in patients with previous IgE sensitization to penicillins

A total of 29 challenges with β-lactam antibiotics were performed in 22 patients with previous positive specific IgE to penicillin and negative skin tests. Four different patterns were seen when evaluating the clinical reaction to challenge (positive/negative) and post-challenge boost of s-IgE (yes/no). Eight patients had negative clinical reaction to challenge and negative post-challenge s-IgE, 8 patients had negative challenge, but positive post-challenge specific IgE levels. Three patients had positive clinical reaction to challenge and positive post-challenge s-IgE whereas 3 patients were challenge positive, but had negative post-challenge s-IgE. The six cases with positive challenge should be classified as allergic to penicillin and the 8 cases with negative challenge and negative post-challenge s-IgE should be classified as not being allergic. It is difficult to decide how the 8 patients with negative challenge and subsequent positive post-challenge s-IgE should be classified and they should probably be considered as still being allergic. The appearance of s-IgE after the challenge may be interpreted as a booster reaction. A conclusion can, however, not be drawn without actually challenging the patients once again. Patients with negative s-IgE at the time of investigation and a case history of reaction several years prior to investigation may have been s-IgE positive at the time of reaction. The 22 patients in the present study would have been classified as s-IgE negative if investigated in average 2.7 years after the clinical reaction took place (range 0–8 years). The frequency of positive outcomes of penicillin challenge in the present study (6/29 challenges – 20.7%) was similar to the findings in our previous study [2], where all patients challenged had a positive history but negative specific IgE to penicillin. Out of 291 challenges 50 had a positive reaction (17.2%) despite that the patients in the present study had previous IgE sensitization.

#### Positive and negative predictive value of serum specific IgE in patients with a history of penicillin allergy and negative skin tests

Several studies have evaluated [7, 15–20] the positive and negative predictive value of s-IgE in patients with a positive history of penicillin allergy but negative skin tests by performing oral/i.v. challenge test. Silva et al. [7] found that only 2 of 7 patients with positive s-IgE had a positive reaction to a challenge test, both were IR with cutaneous symptoms, indicating that a positive s-IgE in patients with a history of penicillin hypersensitivity and negative skin test is not sufficient for confirming the diagnosis. In the same study 2 of 15 patients with negative s-IgE had a positive reaction after challenge, giving a positive predictive value (PPV) of 29% (2/7) and a negative predictive value (NPV) of 89% (13/15). In another study Macy et al. [15] challenged four patients with positive s-IgE (skin test negative), all with negative outcome. Aberer et al. [16–18] stated that detection of IgE antibodies to penicillin does prove sensitization, but not the existence of a clinical allergy. Torres et al. [19] who studied 40 patients with positive s-IgE and negative skin test, found however that 24 patients had a positive IR (immediate reaction) to penicillin during challenge. Blanca et al. [20] challenged 3 s-IgE positive, skin test negative patients with a positive reaction in two only. Both authors describe a high predictive value of a positive s-IgE indicating that challenging patients with current s-IgE will increase the possibility of a positive outcome. This in contrast to our findings, when challenging presently negative but previously IgE positive patients.

#### Immediate reactions (IR)/Nonimmediate reactions (NIR)

Measurement of s-IgE is recommended in patients with a history of an immediate reaction (IR) [3]. Due to the low predictive value of the patient history, [2] we recommend both IR and NIR to be evaluated using the same diagnostic work-up. In this study all 7 patients with IR in case history ended up with positive post-challenge s-IgE, but this was also the case in 4 of the 15 patients with NIR in case history (Table 1). Only 2 respective 1 were challenge positive. S-IgE to penicillin should therefore be measured in all patients with suspected penicillin allergy also since many patients are unable to give a detailed case history regarding time interval between dosage and reaction [21, 22] and further due to the fact that s-IgE both can be positive in challenge proven immediate as well as nonimmediate responders. Ideally, due to the short T½ in many patients, s-IgE should be measured within 6 months after a reaction to penicillin occurred.

#### The significance of positive specific IgE to penicillin

In a study from 2013 Johansson et al. [23] indicated that false-positive penicillin immunoassays constitute an unnoticed, but rather common problem. They found that 26% of patients with a suspected IgE-mediated reaction to penicillin and a positive ImmunoCAP might have specific IgE antibodies to the clinically irrelevant phenylethylamine with a benzyl group (PEA) instead. This may be an argument for measurement of PEA antibodies and for the confirmation of an allergy by clinical tests. Three studies [23–25] have stated that very low levels of allergen s-IgE should be evaluated with caution when serum level of total IgE is above 500 kU/l and that the specificity of tests for anti-β-lactam IgE antibodies declines progressively with increase of total serum IgE. Total serum IgE values were measured in 18 patients (Table 1). Only 3 had total IgE values above 500 kU/l (8 exceeding 150 kU/l). The patient with the highest level of total serum IgE (1773 kU/l) was challenge positive, whereas the others were negative, supporting the theory that elevated level of total IgE may interfere with measurements of specific IgE to penicillins. No significant difference between the patients with high and normal total IgE regarding atopy was found (p > 0.05), data not shown. Kraft et al. [12] have also shown that patients with acute allergic reactions to penicillin have high levels of total serum IgE and that it subsequent decreases in tune with the decline of specific IgE to penicillins.

## Conclusion

An IgE response to penicillin is often a transient phenomenon. In this study approximately half of the patients had a serum half-life of specific IgE to penicillin of less than a year, suggesting that the majority of patients with IgE sensitization at the time of reaction have converted to IgE negativity if measured more than a year after the incident took place. This is supported by the finding that we found the same frequency of positive outcomes during penicillin challenge in the present study compared to our previous study, where all patients (with a case history of penicillin allergy) challenged never had had demonstrable IgE to penicillin [2] in contrast to the present study where patients had previous IgE sensitization. *Regarding the challenges with penicillin in patients with a previous but not present positive s-IgE, a negative sIgE was not associated with tolerance in 63.6% of the tested patients and even a tolerated readministration of penicillins (also during iatrogenic diagnostic workup) may lead to boostering of sIgE with unknown significance, but possibly reoccurrence of penicillin allergy.* Further the more indistinct outcomes in the patients with negative challenge but positive post-challenge s-IgE, no firm conclusion can be drawn without challenging the patients once again. Our results thus suggest that a negative outcome of the 7-day oral challenge should be followed by measurement of specific IgE 4 weeks later, and only if this proved negative, the patient should be classified as tolerant to penicillin.

## Abbreviations

AMP: Ampicillin
AX: Amoxicillin
b.i.d: Twice a day
Β-lactam: Betalactam antibiotic
DX: Dicloxacillin
ICT: Intracutaneous
IE: International units
IgE: Immunoglobulin E
ImmunoCap: Specific immunoglobulin E blood test (Pharmacia Diagnostics AB, Uppsala, Sweden)
IR: Immediate reaction
i.v: Intravenous
NIR: Nonimmediate reaction
NPV: Negative predictive value
P: p-value: Significance level
PEA: Phenylethylamine with a benzyl group
Pen G: Benzylpenicillin
Pen V: Phenoxymethylpenicillin
p.o.7: 7-day oral challenge
PPV: Positive predictive value
s-IgE: Serum specific immunoglobulin E to penicillins
SPT: Standard skin prick test
T½: Serum elimination half-life
t.i.d: Three times a day.
